# Supplementary material for: Ligands of Adrenergic Receptors: A Structural Point of View
Source: Biomolecules. 2021 Jun 24;11(7):936. doi: 10.3390/biom11070936 (PMC8301793; doi:10.3390/biom11070936)
Supplement: Supplementary file 1 [file biomolecules-11-00936-s001.zip › biomolecules-1253862-supplementary.pdf]

Table S1. Representative ligands used in structural studies of adrenergic receptors.

Compound name	Structural formula	Status	Representative PDB	Function	Selectivity[a]
Epinephrine ((–)-adrenaline)	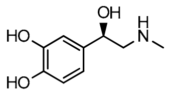	Approved drug	β_2: 4LDO (Nb)[1]<br>β_1: 7BTS (human, Nb)[2]	Agonist	α_1, α_2, β (see Figure 1 for detail)
Norepinephrine ((–)-noradrenaline)	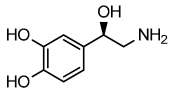	Approved drug	β_1: 7BU6 (human, Nb)[2]	Agonist	α_1, α_2, β (see Figure 1 for detail)
Isoproterenol (isoprenaline)	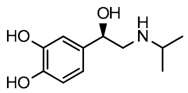	Approved drug	β_2: 7DHR (G_s)[3]<br>β_1: 7JJO (G_s)[4], 7BU7 (human, Nb)[2], 6H7J (Nb)[5], 2Y03[6]	Agonist	β [7-9]
BI-167107	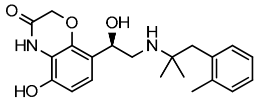	Research	β_2: 3SN6[10] (G_s), 3P0G (Nb)[11]<br>β_1: 7BU7 (human, Nb)[2]	Agonist (arrestin-biased[12])	β (no data for subtype selectivity)
Formoterol	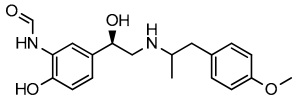	Approved drug	β_2: 7BZ2 (G_s)[13]<br>β_1: 6TKO (arrestin), 6IBL (Nb)[14]	Agonist (arrestin-biased[15])	$\beta_2 > \beta_1$, β_3 [9,16,17]
Isoetharine	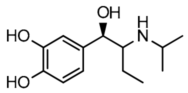	Approved drug	/	Agonist (arrestin-biased in β_2AR[18], G_s-biased in β_1AR[19])	β_1, β_2 [19,20] (no data for β_3AR)
Salmeterol	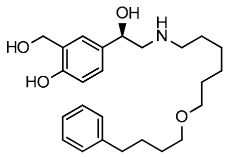	Approved drug	β_2: 6MXT(Nb)[21]	Partial agonist (G_s-biased)	β_2 [9,22]
Salbutamol	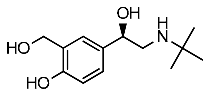	Approved drug	β_2: 7DHI (G_s)[3]<br>β_1: 6H7M (Nb), 2Y04[6]	Partial agonist	$\beta_2 > \beta_1$, β_3 [9,22]
Xamoterol (ICI-118587)	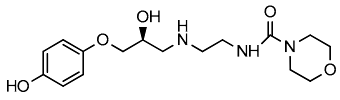	Approved drug	β_1: 6H7N (Nb)[5]	Partial agonist	$\beta_1 > \beta_2 > \beta_3$ [9,22]

Compound name	Structural formula	Status	Representative PDB	Function	Selectivity[a]
Dobutamine	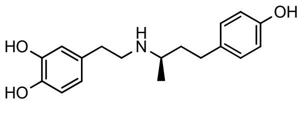	Approved drug	β_1: 2Y00[6], 2Y01[6]	Partial agonist	β [9,16,23]; enantiomer is selective for α_1 [24,25]
Clenbuterol	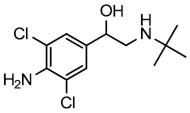	Research	/	Partial agonist	$\beta_2 > \beta_1 > \beta_3$ [9]
Carvedilol	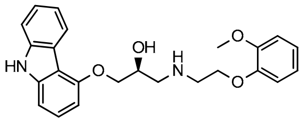	Approved drug	β_2: 6PS3[26]<br>β_1: 4AMJ[27]	G_s antagonist & arrestin agonist[28]	β [22]
Bucindolol	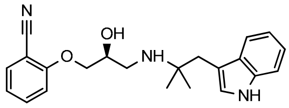	Clinical trial phase 2	β_1: 4AMI[27]	G_s antagonist & arrestin agonist[29]; antagonist of α_1ARs	β_1, $\beta_2 > \beta_3$ [9]; α_1 [30,31]
FAUC50	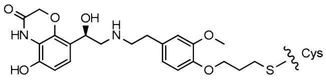	Research	β_2: 3PDS[32]	Covalent agonist	β (no data for subtype selectivity)
'2' [33]	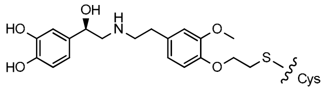	Research	β_2: 4QKX[33]	Covalent agonist	β (no data for subtype selectivity)
Carazolol	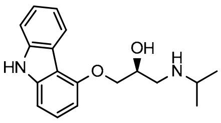	Approved drug	β_2: 2RH1[34]<br>β_1: 7BVQ (human, Nb)[2], 2YCW[35]	Inverse agonist of β_1 & β_2; agonist of β_3 [36]	β_1, $\beta_2 > \beta_3$ [9]
Timolol	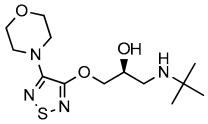	Approved drug	β_2: 3D4S[37], 6PS1[26], 6PS6[26]	Inverse agonist	$\beta_2 > \beta_1 > \beta_3$ [22]
Alprenolol	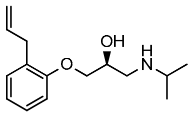	Approved drug	β_2: 3NYA[38], 6PS2[26], 6PRZ[26]	Antagonist	$\beta_2 > \beta_1$, β_3 [22]
Propranolol	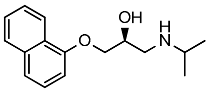	Approved drug	β_2: 6PS5[26]	Antagonist	β_1, $\beta_2 > \beta_3$ [22]
ICI 118551	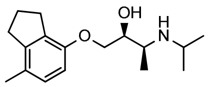	Research	β_2: 3NY8[38], 6PS4[26]	Inverse agonist	$\beta_2 \gg \beta_1$, β_3 [22]

Compound name	Structural formula	Status	Representative PDB	Function	Selectivity[a]
Cyanopindolol	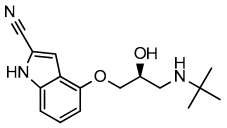	Research	β_1: 6H7O (Nb)[5], 2VT4[39]	Antagonist & weak partial agonist [6H7O]	β_1, β_2 >> β_3 [9]
'2' [38] / '1' [40]	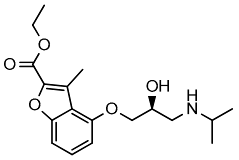	Research (discovered through docking virtural screening[40])	β_2: 3NY9[38]	Inverse agonist	β_2 (no data for other subtypes)
'19' [41]	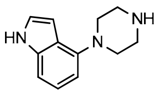	Research (discovered through SPR screening from fragment library[41])	β_1: 3ZPQ[41]	Antagonist	β_1 (no data for other subtypes)
'20' [41]	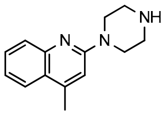	Research (discovered through SPR screening from fragment library[41])	β_1: 3ZPR[41]	Antagonist	β_1 (no data for other subtypes)
Dexmedetomidine	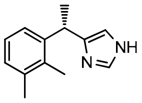	Approved drug	α_{2B}: 6K41 (Gi) , 6K42 (Go)[42]	Partial agonist of α_{2A}; full agonist of α_{2B} & α_{2C}	α_2 [43] [44]
RES	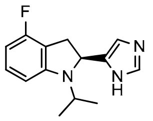	Research	α_{2A}: 6KUY[45]	Partial agonist	α_2 (no data for subtype selectivity)
Clonidine	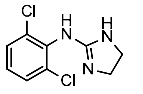	Approved drug	/	Partial agonist	α_2 >> α_1 [43,44]
Guanabenz	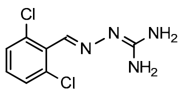	Approved drug	/	Partial agonist of α_{2A} & α_{2B}; antagonist of α_{2C}	α_{2A} > α_{2B}, α_{2C} [44,46]
Guanfacine	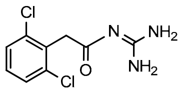	Approved drug	/	Partial agonist	α_2 >> α_1, α_{2A} > α_{2B}, α_{2C} [44,46]
Brimonidine (UK 14,304)	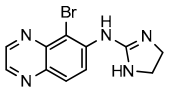	Approved drug	/	Full agonist of α_{2A}; partial agonist of α_{2B} & α_{2C}	α_2 >> α_1 [43,44]

Compound name	Structural formula	Status	Representative PDB	Function	Selectivity[a]
RS 79948	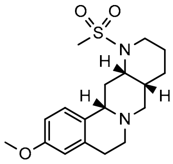	Research	α_{2A}: 6KUX[45]<br>α_{2C}: 6KUW[47]	Antagonist	α_2 [48]
Yohimbine	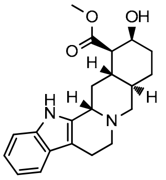	Approved drug	/	Antagonist	α_2 [49]
Tolazoline	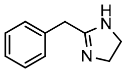	Approved drug	/	Antagonist	$\alpha_{2A} > \alpha_{2B}$, α_{2C} [44]
JP1302	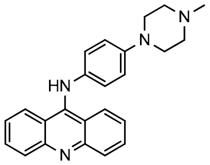	Research	/	Antagonist	$\alpha_{2C} > \alpha_{2A}$, α_{2B} [50]
OPC-28326	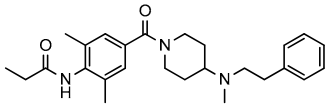	Research	/	Antagonist	$\alpha_{2C} > \alpha_{2A}$, α_{2B} [51]
Phentolamine	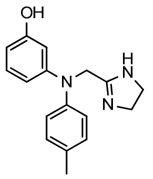	Approved drug	/	Antagonist	α_1, α_2 [49,52]
Prazosin	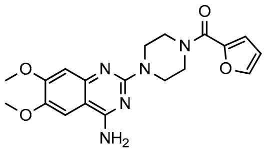	Approved drug	/	Antagonist	$\alpha_1 >> \alpha_2$ [44,53,54]
Ergotamine	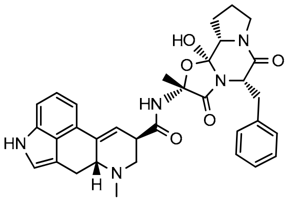	Approved drug	/	Antagonist	$\alpha_2 > \alpha_1 > \beta$ [55]
Cmpd-15PA	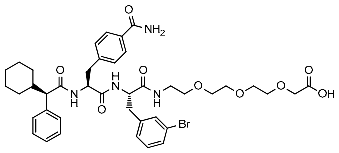	Research	β_2: 5X7D[56]	NAM	β_2 [56]
Cmpd-6FA	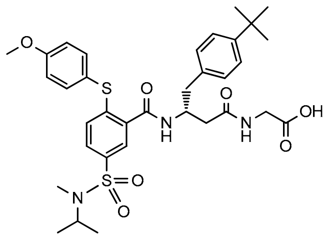	Research	β_2: 6N48 (Nb)[57]	PAM	$\beta_2 > \beta_1$ [57]

Compound name	Structural formula	Status	Representative PDB	Function	Selectivity[a]
AS408	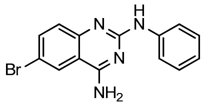	Research	β2: 6OBA[58]	NAM	β2 > β1 [58]

[a]Selective, more than 1000 folds or no effect in unpreferred types/subtypes; >>, 100 to 1000 folds; >, 10 to 100 folds; non-selective, less than 10 folds.

References

1. Ring, A.M.; Manglik, A.; Kruse, A.C.; Enos, M.D.; Weis, W.I.; Garcia, K.C.; Kobilka, B.K. Adrenaline-activated structure of beta2-adrenoceptor stabilized by an engineered nanobody. *Nature* **2013**, *502*, 575-579.
2. Xu, X.; Kaindl, J.; Clark, M.J.; Hubner, H.; Hirata, K.; Sunahara, R.K.; Gmeiner, P.; Kobilka, B.K.; Liu, X. Binding pathway determines norepinephrine selectivity for the human beta1AR over beta2AR. *Cell Res.* **2021**, *31*, 569-579.
3. Yang, F.; Ling, S.; Zhou, Y.; Zhang, Y.; Lv, P.; Liu, S.; Fang, W.; Sun, W.; Hu, L.A.; Zhang, L.; et al. Different conformational responses of the β2-adrenergic receptor-Gs complex upon binding of the partial agonist salbutamol or the full agonist isoprenaline. *Natl Sci Rev* **2020**, nwaa284.
4. Su, M.; Zhu, L.; Zhang, Y.; Paknejad, N.; Dey, R.; Huang, J.; Lee, M.Y.; Williams, D.; Jordan, K.D.; Eng, E.T.; et al. Structural Basis of the Activation of Heterotrimeric Gs-Protein by Isoproterenol-Bound beta1-Adrenergic Receptor. *Mol Cell* **2020**, *80*, 59-71 e54.
5. Warne, T.; Edwards, P.C.; Dore, A.S.; Leslie, A.G.W.; Tate, C.G. Molecular basis for high-affinity agonist binding in GPCRs. *Science* **2019**, *364*, 775-778.
6. Warne, T.; Moukhametzianov, R.; Baker, J.G.; Nehme, R.; Edwards, P.C.; Leslie, A.G.; Schertler, G.F.; Tate, C.G. The structural basis for agonist and partial agonist action on a beta(1)-adrenergic receptor. *Nature* **2011**, *469*, 241-244.
7. Macchia, B.; Balsamo, A.; Breschi, M.C.; Lapucci, A.; Lucacchini, A.; Macchia, F.; Manera, C.; Martinelli, A.; Martini, C.; Martinotti, E.; et al. Conformational effects on the activity of drugs. 13. A revision of previously proposed models for the activation of alpha- and beta-adrenergic receptors. *J. Med. Chem.* **1992**, *35*, 1009-1018.
8. Hoffmann, C.; Leitz, M.R.; Oberdorf-Maass, S.; Lohse, M.J.; Klotz, K.N. Comparative pharmacology of human beta-adrenergic receptor subtypes--characterization of stably transfected receptors in CHO cells. *Naunyn Schmiedebergs Arch Pharmacol* **2004**, *369*, 151-159.
9. Baker, J.G. The selectivity of beta-adrenoceptor agonists at human beta1-, beta2- and beta3-adrenoceptors. *Br J Pharmacol* **2010**, *160*, 1048-1061.
10. Rasmussen, S.G.; DeVree, B.T.; Zou, Y.; Kruse, A.C.; Chung, K.Y.; Kobilka, T.S.; Thian, F.S.; Chae, P.S.; Pardon, E.; Calinski, D.; et al. Crystal structure of the beta2 adrenergic receptor-Gs protein complex. *Nature* **2011**, *477*, 549-555.
11. Rasmussen, S.G.; Choi, H.J.; Fung, J.J.; Pardon, E.; Casarosa, P.; Chae, P.S.; Devree, B.T.; Rosenbaum, D.M.; Thian, F.S.; Kobilka, T.S.; et al. Structure of a nanobody-stabilized active state of the beta(2) adrenoceptor. *Nature* **2011**, *469*, 175-180.
12. Weiss, D.R.; Ahn, S.; Sassano, M.F.; Kleist, A.; Zhu, X.; Strachan, R.; Roth, B.L.; Lefkowitz, R.J.; Shoichet, B.K. Conformation guides molecular efficacy in docking screens of activated beta-2 adrenergic G protein coupled receptor. *ACS Chem Biol* **2013**, *8*, 1018-1026.
13. Zhang, Y.; Yang, F.; Ling, S.; Lv, P.; Zhou, Y.; Fang, W.; Sun, W.; Zhang, L.; Shi, P.; Tian, C. Single-particle cryo-EM structural studies of the beta2AR-Gs complex bound with a full agonist formoterol. *Cell Discov* **2020**, *6*, 45.
14. Lee, Y.; Warne, T.; Nehme, R.; Pandey, S.; Dwivedi-Agnihotri, H.; Chaturvedi, M.; Edwards, P.C.; Garcia-Nafria, J.; Leslie, A.G.W.; Shukla, A.K.; et al. Molecular basis of beta-arrestin coupling to formoterol-bound beta1-adrenoceptor. *Nature* **2020**, *583*, 862-866.
15. Rajagopal, S.; Ahn, S.; Rominger, D.H.; Gowen-MacDonald, W.; Lam, C.M.; Dewire, S.M.; Violin, J.D.; Lefkowitz, R.J. Quantifying ligand bias at seven-transmembrane receptors. *Mol. Pharmacol.* **2011**, *80*, 367-377.
16. Isogaya, M.; Sugimoto, Y.; Tanimura, R.; Tanaka, R.; Kikkawa, H.; Nagao, T.; Kurose, H. Binding pockets of the beta(1)- and beta(2)-adrenergic receptors for subtype-selective agonists. *Mol. Pharmacol.* **1999**, *56*, 875-885.
17. Jacobsen, J.R.; Aggen, J.B.; Church, T.J.; Klein, U.; Pfeiffer, J.W.; Pulido-Rios, T.M.; Thomas, G.R.; Yu, C.; Moran, E.J. Multivalent design of long-acting beta(2)-adrenoceptor agonists incorporating biarylamines. *Bioorg. Med. Chem. Lett.* **2014**, *24*, 2625-2630.
18. Drake, M.T.; Violin, J.D.; Whalen, E.J.; Wisler, J.W.; Shenoy, S.K.; Lefkowitz, R.J. beta-arrestin-biased agonism at the beta2-adrenergic receptor. *J. Biol. Chem.* **2008**, *283*, 5669-5676.
19. Lukasheva, V.; Devost, D.; Le Gouill, C.; Namkung, Y.; Martin, R.D.; Longpre, J.M.; Amraei, M.; Shinjo, Y.; Hogue, M.; Lagace, M.; et al. Signal profiling of the beta1AR reveals coupling to novel signalling pathways and distinct phenotypic responses mediated by beta1AR and beta2AR. *Sci Rep* **2020**, *10*, 8779.
20. Peterson, Y.K.; Cameron, R.B.; Wills, L.P.; Trager, R.E.; Lindsey, C.C.; Beeson, C.C.; Schnellmann, R.G. beta2-Adrenoceptor

agonists in the regulation of mitochondrial biogenesis. *Bioorg. Med. Chem. Lett.* **2013**, *23*, 5376-5381.
21. Masureel, M.; Zou, Y.; Picard, L.P.; van der Westhuizen, E.; Mahoney, J.P.; Rodrigues, J.; Mildorf, T.J.; Dror, R.O.; Shaw, D.E.; Bouvier, M.; et al. Structural insights into binding specificity, efficacy and bias of a beta2AR partial agonist. *Nat. Chem. Biol.* **2018**, *14*, 1059-1066.
22. Baker, J.G. The selectivity of beta-adrenoceptor antagonists at the human beta1, beta2 and beta3 adrenoceptors. *Br J Pharmacol* **2005**, *144*, 317-322.
23. Horinouchi, T.; Koike, K. Further characterization of beta3-adrenoceptors in the guinea pig gastric fundus: stereoselectivity, beta-adrenoceptor alkylation, and structure-activity relationship. *Can J Physiol Pharmacol* **2001**, *79*, 985-995.
24. Ruffolo, R.R., Jr.; Spradlin, T.A.; Pollock, G.D.; Waddell, J.E.; Murphy, P.J. Alpha and beta adrenergic effects of the stereoisomers of dobutamine. *J. Pharmacol. Exp. Ther.* **1981**, *219*, 447-452.
25. Waldeck, B. "The beta1-selective adrenoceptor agonist dobutamine": a fallacy being perpetuated. *Chirality* **2011**, *23*, 63-64.
26. Ishchenko, A.; Stauch, B.; Han, G.W.; Batyuk, A.; Shiriaeva, A.; Li, C.; Zatsepin, N.; Weierstall, U.; Liu, W.; Nango, E.; et al. Toward G protein-coupled receptor structure-based drug design using X-ray lasers. *IUCrJ* **2019**, *6*, 1106-1119.
27. Warne, T.; Edwards, P.C.; Leslie, A.G.; Tate, C.G. Crystal structures of a stabilized beta1-adrenoceptor bound to the biased agonists bucindolol and carvedilol. *Structure* **2012**, *20*, 841-849.
28. Wisler, J.W.; DeWire, S.M.; Whalen, E.J.; Violin, J.D.; Drake, M.T.; Ahn, S.; Shenoy, S.K.; Lefkowitz, R.J. A unique mechanism of beta-blocker action: carvedilol stimulates beta-arrestin signaling. *Proc Natl Acad Sci U S A* **2007**, *104*, 16657-16662.
29. Galandrin, S.; Oligny-Longpre, G.; Bonin, H.; Ogawa, K.; Gales, C.; Bouvier, M. Conformational rearrangements and signaling cascades involved in ligand-biased mitogen-activated protein kinase signaling through the beta1-adrenergic receptor. *Mol Pharmacol* **2008**, *74*, 162-172.
30. Watts, S.W.; Fink, G.D.; Silver, P.J.; Cushing, D.J. Interaction of the beta adrenergic receptor antagonist bucindolol with serotonergic receptors. *J Cardiovasc Pharmacol* **2000**, *35*, 29-36.
31. Marwood, J.F.; Stokes, G.S. Bucindolol has serotonin and alpha-adrenoceptor blocking properties. *J Cardiovasc Pharmacol* **1985**, *7 Suppl 7*, S67-69.
32. Rosenbaum, D.M.; Zhang, C.; Lyons, J.A.; Holl, R.; Aragao, D.; Arlow, D.H.; Rasmussen, S.G.; Choi, H.J.; Devree, B.T.; Sunahara, R.K.; et al. Structure and function of an irreversible agonist-beta(2) adrenoceptor complex. *Nature* **2011**, *469*, 236-240.
33. Weichert, D.; Kruse, A.C.; Manglik, A.; Hiller, C.; Zhang, C.; Hubner, H.; Kobilka, B.K.; Gmeiner, P. Covalent agonists for studying G protein-coupled receptor activation. *Proc Natl Acad Sci U S A* **2014**, *111*, 10744-10748.
34. Cherezov, V.; Rosenbaum, D.M.; Hanson, M.A.; Rasmussen, S.G.; Thian, F.S.; Kobilka, T.S.; Choi, H.J.; Kuhn, P.; Weis, W.I.; Kobilka, B.K.; et al. High-resolution crystal structure of an engineered human beta2-adrenergic G protein-coupled receptor. *Science* **2007**, *318*, 1258-1265.
35. Moukhametzianov, R.; Warne, T.; Edwards, P.C.; Serrano-Vega, M.J.; Leslie, A.G.; Tate, C.G.; Schertler, G.F. Two distinct conformations of helix 6 observed in antagonist-bound structures of a beta1-adrenergic receptor. *Proc Natl Acad Sci U S A* **2011**, *108*, 8228-8232.
36. Mejean, A.; Guillaume, J.L.; Strosberg, A.D. Carazolol: a potent, selective beta 3-adrenoceptor agonist. *Eur. J. Pharmacol.* **1995**, *291*, 359-366.
37. Hanson, M.A.; Cherezov, V.; Griffith, M.T.; Roth, C.B.; Jaakola, V.P.; Chien, E.Y.; Velasquez, J.; Kuhn, P.; Stevens, R.C. A specific cholesterol binding site is established by the 2.8 A structure of the human beta2-adrenergic receptor. *Structure* **2008**, *16*, 897-905.
38. Wacker, D.; Fenalti, G.; Brown, M.A.; Katritch, V.; Abagyan, R.; Cherezov, V.; Stevens, R.C. Conserved binding mode of human beta2 adrenergic receptor inverse agonists and antagonist revealed by X-ray crystallography. *J. Am. Chem. Soc.* **2010**, *132*, 11443-11445.
39. Warne, T.; Serrano-Vega, M.J.; Baker, J.G.; Moukhametzianov, R.; Edwards, P.C.; Henderson, R.; Leslie, A.G.; Tate, C.G.; Schertler, G.F. Structure of a beta1-adrenergic G-protein-coupled receptor. *Nature* **2008**, *454*, 486-491.
40. Kolb, P.; Rosenbaum, D.M.; Irwin, J.J.; Fung, J.J.; Kobilka, B.K.; Shoichet, B.K. Structure-based discovery of beta2-adrenergic receptor ligands. *Proc Natl Acad Sci U S A* **2009**, *106*, 6843-6848.
41. Christopher, J.A.; Brown, J.; Dore, A.S.; Errey, J.C.; Koglin, M.; Marshall, F.H.; Myszka, D.G.; Rich, R.L.; Tate, C.G.; Tehan, B.; et al. Biophysical fragment screening of the beta1-adrenergic receptor: identification of high affinity arylpiperazine leads using structure-based drug design. *J. Med. Chem.* **2013**, *56*, 3446-3455.
42. Yuan, D.; Liu, Z.; Kaindl, J.; Maeda, S.; Zhao, J.; Sun, X.; Xu, J.; Gmeiner, P.; Wang, H.W.; Kobilka, B.K. Activation of the alpha2B adrenoceptor by the sedative sympatholytic dexmedetomidine. *Nat. Chem. Biol.* **2020**, *16*, 507-512.
43. Virtanen, R.; Savola, J.M.; Saano, V.; Nyman, L. Characterization of the selectivity, specificity and potency of medetomidine as an alpha 2-adrenoceptor agonist. *Eur. J. Pharmacol.* **1988**, *150*, 9-14.
44. Jasper, J.R.; Lesnick, J.D.; Chang, L.K.; Yamanishi, S.S.; Chang, T.K.; Hsu, S.A.; Daunt, D.A.; Bonhaus, D.W.; Eglen, R.M. Ligand efficacy and potency at recombinant alpha2 adrenergic receptors: agonist-mediated [35S]GTPgammaS binding. *Biochem Pharmacol* **1998**, *55*, 1035-1043.
45. Qu, L.; Zhou, Q.; Xu, Y.; Guo, Y.; Chen, X.; Yao, D.; Han, G.W.; Liu, Z.J.; Stevens, R.C.; Zhong, G.; et al. Structural Basis of the Diversity of Adrenergic Receptors. *Cell Rep* **2019**, *29*, 2929-2935 e2924.
46. Takeuchi, K.; Kogure, M.; Hashimoto, T. Comparison of agonistic and antagonistic actions of guanabenz and guanfacin on

alpha 1 and alpha 2-adrenoceptors in isolated smooth muscles. *Jpn J Pharmacol* **1987**, *43*, 267-275.
47. Chen, X.; Xu, Y.; Qu, L.; Wu, L.; Han, G.W.; Guo, Y.; Wu, Y.; Zhou, Q.; Sun, Q.; Chu, C.; et al. Molecular Mechanism for Ligand Recognition and Subtype Selectivity of alpha2C Adrenergic Receptor. *Cell Rep* **2019**, *29*, 2936-2943 e2934.
48. Milligan, C.M.; Linton, C.J.; Patmore, L.; Gillard, N.; Ellis, G.J.; Towers, P. [3H]-RS-79948-197, a high affinity radioligand selective for alpha 2-adrenoceptor subtypes. *Ann. N. Y. Acad. Sci.* **1997**, *812*, 176-177.
49. Devedjian, J.C.; Esclapez, F.; Denis-Pouxviel, C.; Paris, H. Further characterization of human alpha 2-adrenoceptor subtypes: [3H]RX821002 binding and definition of additional selective drugs. *Eur. J. Pharmacol.* **1994**, *252*, 43-49.
50. Sallinen, J.; Hoglund, I.; Engstrom, M.; Lehtimaki, J.; Virtanen, R.; Sirvio, J.; Wurster, S.; Savola, J.M.; Haapalinna, A. Pharmacological characterization and CNS effects of a novel highly selective alpha2C-adrenoceptor antagonist JP-1302. *Br J Pharmacol* **2007**, *150*, 391-402.
51. Sun, B.; Lockyer, S.; Li, J.; Chen, R.; Yoshitake, M.; Kambayashi, J.I. OPC-28326, a selective femoral vasodilator, is an alpha2C-adrenoceptor-selective antagonist. *J Pharmacol Exp Ther* **2001**, *299*, 652-658.
52. Shibata, K.; Foglar, R.; Horie, K.; Obika, K.; Sakamoto, A.; Ogawa, S.; Tsujimoto, G. KMD-3213, a novel, potent, alpha 1a-adrenoceptor-selective antagonist: characterization using recombinant human alpha 1-adrenoceptors and native tissues. *Mol. Pharmacol.* **1995**, *48*, 250-258.
53. Ford, A.P.; Daniels, D.V.; Chang, D.J.; Gever, J.R.; Jasper, J.R.; Lesnick, J.D.; Clarke, D.E. Pharmacological pleiotropism of the human recombinant alpha1A-adrenoceptor: implications for alpha1-adrenoceptor classification. *Br J Pharmacol* **1997**, *121*, 1127-1135.
54. Williams, T.J.; Blue, D.R.; Daniels, D.V.; Davis, B.; Elworthy, T.; Gever, J.R.; Kava, M.S.; Morgans, D.; Padilla, F.; Tassa, S.; et al. In vitro alpha1-adrenoceptor pharmacology of Ro 70-0004 and RS-100329, novel alpha1A-adrenoceptor selective antagonists. *Br J Pharmacol* **1999**, *127*, 252-258.
55. Peng, Y.; McCorvy, J.D.; Harpsoe, K.; Lansu, K.; Yuan, S.; Popov, P.; Qu, L.; Pu, M.; Che, T.; Nikolajsen, L.F.; et al. 5-HT2C Receptor Structures Reveal the Structural Basis of GPCR Polypharmacology. *Cell* **2018**, *172*, 719-730 e714.
56. Liu, X.; Ahn, S.; Kahsai, A.W.; Meng, K.C.; Latorraca, N.R.; Pani, B.; Venkatakrishnan, A.J.; Masoudi, A.; Weis, W.I.; Dror, R.O.; et al. Mechanism of intracellular allosteric beta2AR antagonist revealed by X-ray crystal structure. *Nature* **2017**, *548*, 480-484.
57. Liu, X.; Masoudi, A.; Kahsai, A.W.; Huang, L.Y.; Pani, B.; Staus, D.P.; Shim, P.J.; Hirata, K.; Simhal, R.K.; Schwalb, A.M.; et al. Mechanism of beta2AR regulation by an intracellular positive allosteric modulator. *Science* **2019**, *364*, 1283-1287.
58. Liu, X.; Kaindl, J.; Korczynska, M.; Stossel, A.; Dengler, D.; Stanek, M.; Hubner, H.; Clark, M.J.; Mahoney, J.; Matt, R.A.; et al. An allosteric modulator binds to a conformational hub in the beta2 adrenergic receptor. *Nat. Chem. Biol.* **2020**, *16*, 749-755.
